# Supplementary figures and images for: Syn-COM: A Multi-Level Predictive Synergy Framework for Innovative Drug Combinations
Source: Pharmaceuticals (Basel). 2024 Sep 18;17(9):1230. doi: 10.3390/ph17091230 (PMC11434649; doi:10.3390/ph17091230)

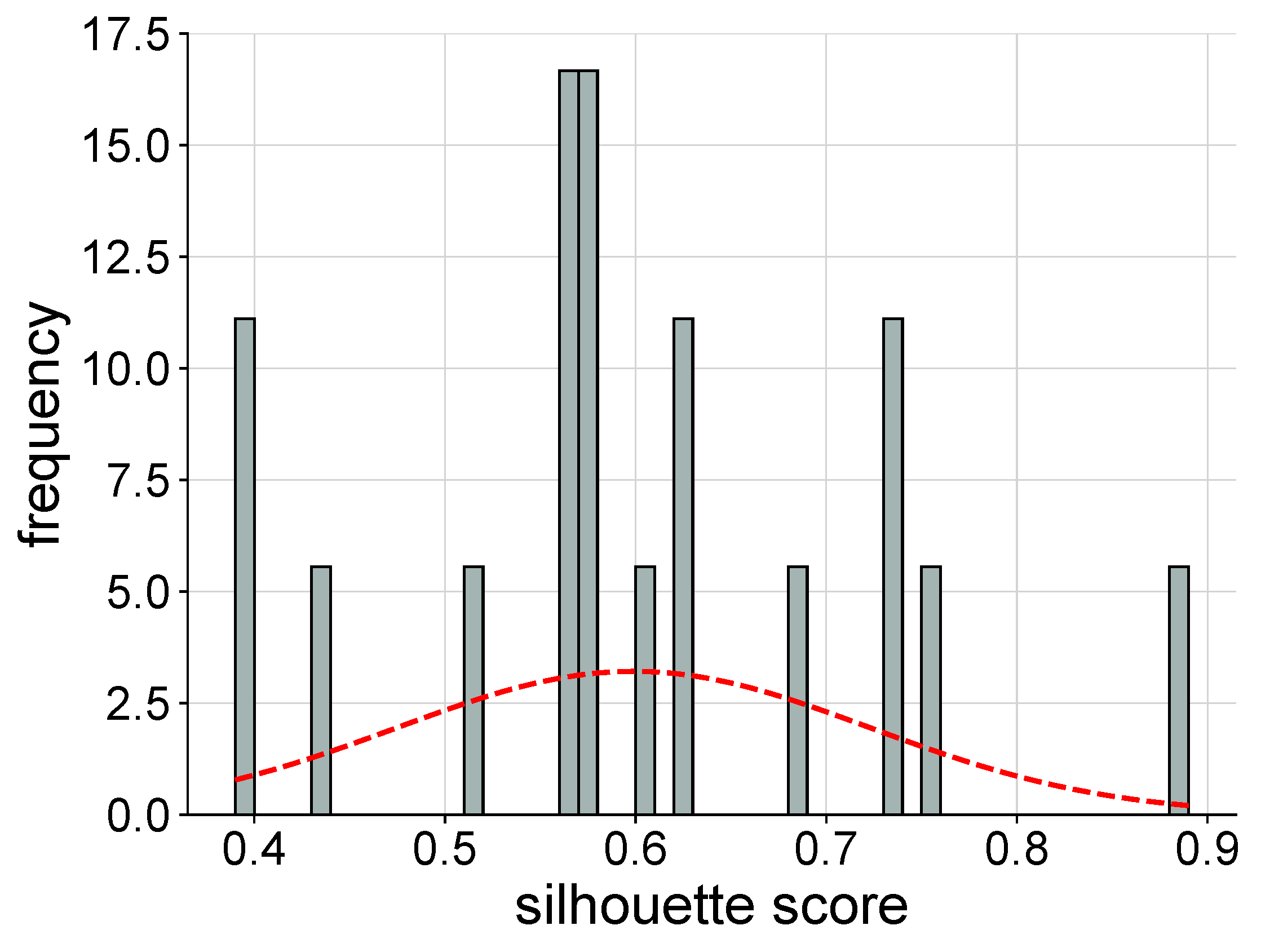

Supplement: Supplementary file 1 [file pharmaceuticals-17-01230-s001.zip › FigureS1. silhouette score.jpg]

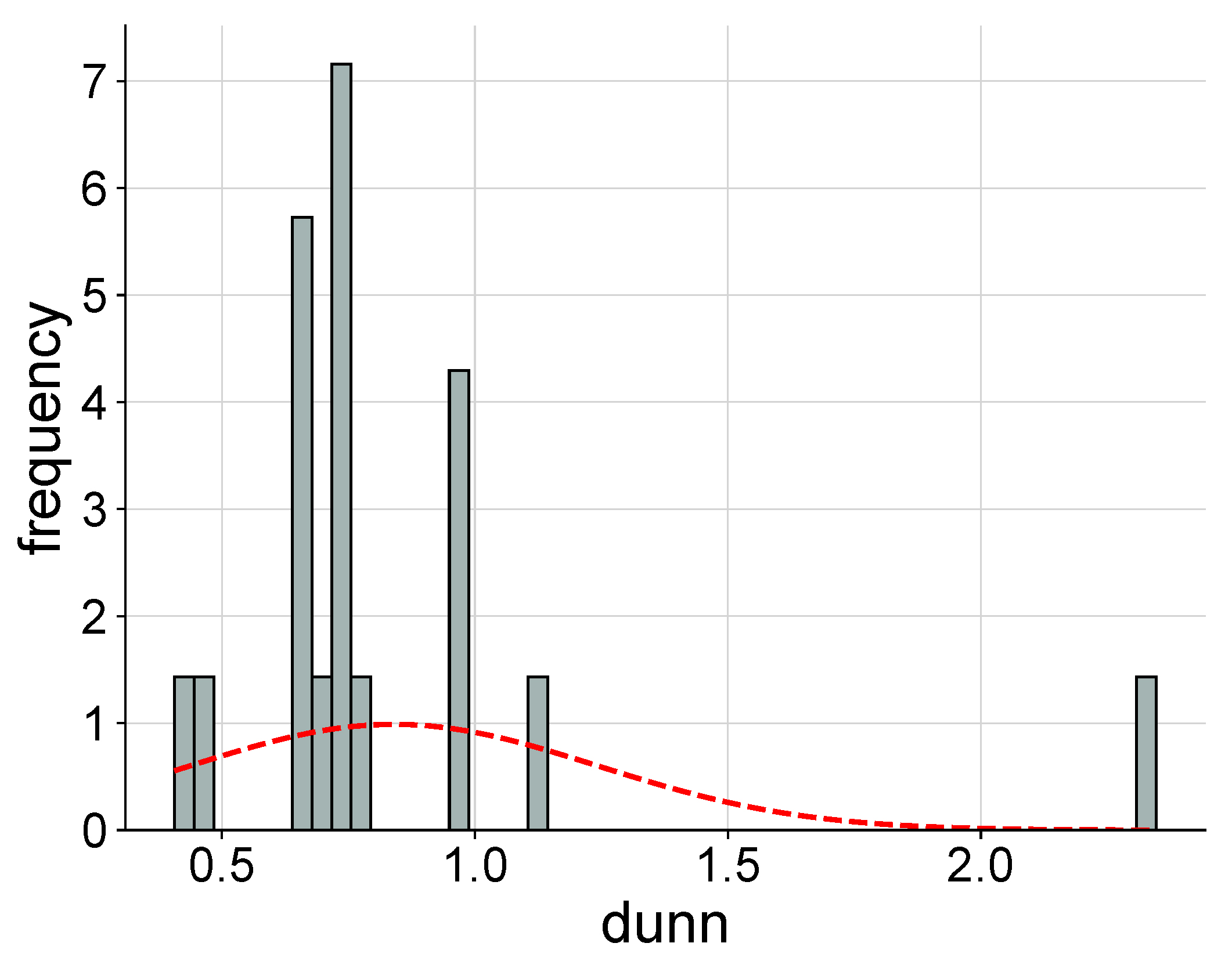

Supplement: Supplementary file 1 [file pharmaceuticals-17-01230-s001.zip › FigureS2. Dunn index.jpg]
